# Harvard Odontological Society

**Published:** 1891-11

**Authors:** 

**Affiliations:** Harvard Odontological Society


### HARVARD ODONTOLOGICAL SOCIETY.

The Harvard Odontological Society held its regular monthly meeting, April 30, 1891, at Young's Hotel, Boston. The President, Dr. J. E. Stanton, in the chair. Dr. Harold C. Ernst was the speaker (impromptu) of the evening. Subject: "A Few Words in Regard to the Sterilization of Instruments."

(For Dr. Ernst's paper, see page 741.)

*Dr. Potter.*—This is a subject about which I have given considerable thought from the very first of my professional work. It has, however, always been somewhat perplexing, even though consulting the current authorities, to be quite sure as to what processes or chemicals had a true germicidal action. And I am particularly pleased to-night in getting so much reliable information direct from the laboratory. For it is upon laboratory experiments that we must rely if we want to know with certainty as to the value of germicides. As to Dr. Ernst's methods, I have in a way practised them for some time. I should like to ask him a few questions. In the first place, What is the temperature of the Arnold sterilizer? If the steam is to any extent kept confined, the heat must rise above 100° C. Secondly, after the steam is up, how long a time must instruments be submitted to its action in order to be thoroughly sterilized? Thirdly, would the addition of a potassium solution to the water control to any extent the matter of rust? We know very well that in boiling instruments the addition of liquor potass. prevents their rusting, and my query is, Would the same substance have any influence in an Arnold sterilizer?

My own practice has been in the care of instruments to boil such of them as could not be injured by boiling, and to submit others, as chisels and excavators, to a ninety-five per cent. solution of carbolic acid. Acid of this strength does not rust instruments as do the weaker solutions. The Bunsen burner I have used extensively on certain instruments which could be exposed to its flame without material injury. How to sterilize a mouth-mirror is still an unsolved problem to me.

*Dr. Taft.*—I should like to ask if a thorough cleansing of all instruments with soap and water with a good brush will not accomplish sterilization?

*Dr. Niles.*—I am very much impressed with the necessity of sterilizing our instruments. The difficulty seems to be to get at it conveniently as we are engaged in going from one patient to another. Of course, we may have two sets of instruments, and while operating upon one patient, our assistant can sterilize the instruments that we have used in the preceding case. But it is necessary that this should be done quickly and conveniently near the chair, and the process continually going on. I have thought of getting an oven. An idea has occurred to me to-night that possibly we might simplify the matter by using hot oil, or carbolized oil, or carbolized glycerin. We can attain a higher temperature in these mediums, and the instrument simply dipped for a few moments in a very small vessel, which might be arranged near the chair over a Bunsen lamp, it seems to me would accomplish what is desired. The idea, as I understand it, is to get a sufficiently high temperature to destroy germinal life. Water might boil away; if hot oil be used, we would avoid rusting the instruments.

Another interesting point brought out by the professor is in relation to the germ found in the mouth called the micrococcus of sputum septicæmia. I should like to ask him if these germs are found in every mouth? He speaks of people who have been bitten, and the wounds have been very hard to heal; and I thought possibly this germ may have something to do with the very obstinate affection of the gums usually known as pyorrhœa alveolaris, or Rigg's disease. If these are present in all mouths and in a normal condition of the mouth, is not their presence dangerous, or active in the production of these pus-discharging sockets of teeth?

*Dr. Giblin.*—Is it necessary that there be a lesion—an abrasion of the mucous membrane—in order that bacilli become dangerous in the mouth?

*Dr. Werner.*—To me the mouth-mirror is the instrument *par excellence* accountable for carrying infection from mouth to mouth. Many a dentist will wash his excavators, his chisels, and minor instruments, but the mouth-mirror will be most neglected. What shall we do with it? Will the essayist instruct us as to the germicidal properties of creolin?

*Dr. Stoddard.*—I would like to ask Dr. Ernst if electricity has been used in sterilizing instruments, and if so, how powerful a current is necessary?

*Dr. Potter.*—If alcohol is good for anything as a sterilizer, I have here a mirror which has been for many hours submerged in it without doing any damage.

*Dr. Werner.*—We must not forget our napkins, which may be a great source for conveying disease from one mouth to another. Dental napkins are a great source of annoyance to many cleanly patients, and I presume washing is not a very efficacious way of sterilizing them.

*Dr. Taft.*—If the system is in a condition of perfect health, are not the bacilli of tuberculosis, syphilis, septicæmia, and various other infectious diseases, rendered inoperative? It is well known that we are full of bacteria of one kind or another, and when the system is in a condition of perfect health, we are not aware of their existence to any alarming degree, but the moment the vitality becomes lowered, these bacteria become effective and work injury. Otherwise, why is it not fair to presume that our patients are being constantly inoculated with the various forms of bacilli?

*Dr. Ernst.*—Mr. President, I will, as far as I can, answer the questions in the order in which they have been asked.

Dr. Potter asks about the temperature and the length of time which the Arnold sterilizer should be used, and the effectiveness of a solution of potash for preventing the rust. The temperature obtained is about 101° to 102° C., the steam being slightly under pressure. That pressure is increased if the heat is increased and the steam given off more rapidly, but that is quite enough. The time for exposure for complete sterilization need not and does not extend over fifteen minutes after the steam has begun to pass; twenty minutes gives us an ample margin. The effectiveness of a potash solution is unquestionably very great, and there is no reason why it would not work just as well in the Arnold sterilizer as in any of the ordinary forms of steam-bath.

Of course, in what I have said to the Society this evening I have generalized, because I have realized that the means must vary according to the circumstances, and, on account of the difference in circumstances, no definite lines of procedure can be laid down.

Dr. Potter also spoke of carbolic acid, and, as he says, it is very effective if used in sufficient strength and for a sufficient length of time, having only the disadvantage that other chemicals may have, —the smell,—which renders it objectionable to most patients, and particularly if used in the strength of which Dr. Potter speaks.

The use of the naked flame of the Bunsen burner is as effective as anything can be, provided the instruments can stand it, but there are very few that I know of that can stand a Bunsen flame for a sufficient length of time to complete the sterilization. It would be necessary to heat the instrument almost to a red heat every time, and that, of course, if repeated often, will spoil the temper of any instrument.

Alcohol is not a germicide, it is an antiseptic only. That can be proven by the immersion of small pieces of tubercle in alcohol.

While in the alcohol the bacteria remain inactive, but on removing them and washing with water, they resume their activity. The use of alcohol as I have employed it myself in cleansing instruments—such instruments, for instance, as syringes which have been used for injections—is entirely for washing away traces of carbolic acid,—not at all as a germicide.

Dr. Taft asks why soap and water are not sufficient. It is sufficient for cleaning away dirt, but it is not sufficient for the destruction of bacteria,—they are simply diluted, but not destroyed, and wiping does not remove them with certainty. The same objection is to be brought against cloth, which will polish the instruments more or less, but does not clean the small corners, and any minute particle that may be present there might be sufficient to cause a great deal of harm. The portion that is necessary for the production of pathological change is inconceivably small, and may readily lurk in the corners of the burs of the dental-engine, and require the aid of the lens to see them. The rubber is simply good for polishing, and, like the soap and water, and the cloth, does not remove the bacteria from the interstices of the instrument.

The objection to carbolized oil or glycerin in my experience is that it very soon fills a room with such fumes that it is impossible to live in it. I have attempted to use it in my laboratory, which is a room larger than this one, and found that I would have to open all the windows or go out myself. The effectiveness of sterilization is very perfect. Dr. Niles also spoke of the evaporation of the water. In the Arnold sterilizer the water does not evaporate, so that you can use it all day long without renewing the water. The apparatus is easy of manipulation, and is quite small, so that it can be conveniently employed for your purposes. The question as to the causation of pyorrhœa alveolaris by the micrococcus of sputum septicæmia is a pertinent one, but it is not yet settled. The probabilities are that that form of suppuration is produced by one of the ordinary suppurative bacteria, and not by this micrococcus of sputum septicæmia. The organism does not occur in everybody's mouth and at all times, and yet it is likely to be found at almost any time. Exactly what laws govern its appearance or disappearance are not yet made out.

The question was asked whether an abrasion was necessary for the inoculation by the various bacilli. It is true that an abrasion of the mucous membrane is necessary for the local infection of the patient, but the presence of the rubber dam does not by any means prove that all of the infectious material is excluded from the mouth.

The mouth-mirror seems to me to be an offender, from my own experience. I have suspected, what has been said this evening, that it was not cleaned quite as thoroughly as it should be. One suggestion that I would make is that the handles of the mirrors should be made of a solid piece of steel. The added weight would not count for much, and the ease of taking care of the instrument would more than counterbalance it. They could be sterilized either in the sterilizer or in carbolic acid, but whether that would injure the silver on the glass I do not know. If that be true, it seems to me a matter of very slight mechanical ingenuity to return to the old style of metallic mirrors, and on a small scale I think they could be made quite as effective as the present form.

With regard to the effect of creolin as a germicide, I have not tested it scientifically, and do not know anything about it; that sort of question is one where the dental specialist should come in and investigate for himself. Some member of your Society should come down to my laboratory and settle that question. These are special things, the details of which I cannot make myself personally familiar with, and one of my objects in doing the work that I have done was to have a laboratory which should be thoroughly equipped, and should be open to any one who will take the proper training for doing this special work.

Electricity has not, so far as I know, been used in the disinfection of instruments. Its influence upon the vitality of bacteria in culture has been tried, and is unsettled, and certainly slight. Therefore, I should not suppose it would be of any service for the sterilization of instruments.

The sterilization of napkins can be very easily accomplished in this same Arnold sterilizer. All that you need is to have a wire basket in which they can be placed loosely and left in the sterilizer for fifteen or twenty minutes. Then they can be washed without any danger to anybody and used over and over again.

The question of perfect health and of lowered vitality is an important one in the production of pathological changes in the human organism, but the change of vitality which makes a person susceptible, as transferring him from a condition of insusceptibility, is so extremely delicate that nobody can tell when that change occurs. We know that a perfectly healthy person may be stricken down instantly by almost any form of disease, and all that we can say is that this element of lowered vitality has come into play, but exactly how or when we cannot say.

Referring to the practicability of this question, I have endeavored to be as practical to you as I knew how. It was necessary to speak of the bases of the remarks and to explain them as well as possible. The practical part of it is distinctly what I am interested in as much as you are.

*Dr. Reilly.*—Before the subject is passed, I should like to ask Dr. Ernst if he can suggest anything for the use of the hands or nails which will render them sterile?

*Dr. Ernst.*—There is nothing that is absolutely certain to accomplish that except corrosive sublimate. To many skins that is like pouring kerosene on the kitchen-fire,—it will instantly start up an irritation that is anything but pleasant; but if the skin will stand it, a solution, one to five thousand, used with soap and brush, and followed by pure water, is very effectual. If one's hands cannot stand that, then the best we can do is to clean out the corners of the nails with a fresh orange-wood tooth-pick, and wash the hands as thoroughly as possible in water.

*Dr. Bigelow.*—Dr. Ernst made a remark that corrosive sublimate could be used effectively on instruments; I would like to know what strength is best.

*Dr. Ernst.*—One to one thousand, and it would be a little unsafe to use upon instruments that are going right into the mouth. I have seen injurious effect from absorption when instruments were used so quickly after being immersed.

*Dr. Stanley.*—I understood Dr. Ernst to say that a steam-bath was rather more effective than boiling in water. I cannot see why it should be.

*Dr. Ernst.*—It has been found by experiment that the actual temperature of a water-bath varies from the bottom to the top of a vessel, and you might get thorough disinfection at the bottom next to the flame, and not at the top. In the steam sterilizer the steam comes up and around the instruments, and the temperature is kept up by the constantly-renewed stream of live steam, and this being kept under a slight pressure, the temperature is always the same.

*Dr. Taft.*—It is said that soap and water is not an effectual way of getting rid of the bacteria, and yet it seems to me that we can convey micro-organisms from one mouth to another by our hands as easily as in any other way. Now, if the hands are not subjected to a steam-bath, how are we sure that we get rid of every bacterium? For, as I understand it, one can work as much injury as a thousand. To be sure, we wash our hands and cleanse the finger-nails after every operation before going to another patient, but we do not subject them to a steam-bath nor immerse in germicidal solutions. How, then, do we know when we have gotten rid of all the germs, and when the hands and nails are surgically clean?

*Dr. Ernst.*—In the first place, the cleansing of the finger-nails should be done before the final washing of the hands, and the washing of the hands should be done under tap, so as to carry off everything possible. In the second place, one cannot say that you actually do get rid of all the bacteria, but that you are doing the best you can for the protection of yourselves and your patients.

*Dr. Taft.*—In view of that, how much real danger is there in this whole subject of inoculation?

*Dr. Ernst.*—That is difficult to say. That disease is produced by these organisms is unquestionably true. Personally, I believe that the slightest lesion of the skin may be all that is necessary for the entrance of the bacilli. I was convinced of this by an experiment where I inoculated myself accidentally in my own laboratory. In a short time I had an abscess larger than my hand, from which I made cultures and carried out a scientific experiment. Several medical gentlemen were acquainted with the case and saw the result. The danger may not be very great; that it does occur there is not the slightest question. Some time ago I discovered that a patient of mine, who was afflicted with syphilis and had syphilitic patches in the mucous membrane of the mouth, had gone to a dentist whom I knew did not take the best precautions with his instruments, and I kept the gentleman away from that dentist. I should use my influence with my patients to keep them from a dentist whom I knew neglected the care of his instruments, and would not consider that I was violating the rules of professional courtesy by so doing, but simply protecting my patients.

*Dr. Taft.*—Do I understand, then, that unless there is an abraded surface there is no danger of inoculation?

*Dr. Ernst.*—That is an extremely difficult question to answer. The probabilities are that there is none. At the same time, even if that be true, we could not tell without the aid of the microscope that the entire surface of the mucous membrane is whole. There may be an abrasion sufficient for the entrance of the bacteria, which you saw on the screen, which could not be perceived by a mere inspection, so that even if a lesion be necessary, we cannot say that it is or is not present.

*Dr. Giblin.*—I would like to ask Dr. Ernst if there is a bacillus or bacterium which will cause tonsillitis? A relative of mine was afflicted with it, and while she was ill her little girl was taken down with acute tonsillitis. I thought possibly, by the use of a spoon or some table utensil, which would not be sterilized by the usual method of cleaning, it might have been transmitted.

*Dr. Ernst.*—You were undoubtedly correct in your conclusions. Tonsillitis is unquestionably due to bacterial action ; but, like a number of other diseases, it is not a specific disease,—that is, not all the cases of tonsillitis are produced by the same organism.

HENRY L. UPHAM, D.M.D.,
*Editor Harvard Odontological Society.*

